# Symes on Thymol and Thymol Camphor

**Published:** 1879-08

**Authors:** 


### SYMES ON THYMOL AND THYMOL CAMPHOR.

Dr. Symes, in the Pharmaceutical Journal, publishes the results of his researches on the combination of thymol, chloral hydrate, and camphor, acting as an antiseptic. The two former drugs are rubbed together in a mortar, and an equal quantity of camphor added, which liquefies the whole, and produces a powerful antiseptic. Its virtues were tested on urine containing pus, which was already beginning to decompose. Two drops of the compound were added, and the putrefaction was stopped. If thymol and camphor alone are rubbed together they also become liquid, and this is a convenient form from which to prepare the ointment. Thymol camphor can be mixed in almost any proportion with vaseline, ung. petrolei, or ozokerin, and the thymol will not separate out in crystals, as when thymol alone is used. A solution of thymol in water (one in one thousand) is sufficiently strong for the spray in surgical operations. If used for the throat, milk and glacial acetic acid will be found to be good solvents for it. *Ibid.*

